# Long-Term Survival Among Children With Trisomy 13 and Trisomy 18 by Cytogenetic Status

**DOI:** 10.1001/jamanetworkopen.2025.29885

**Published:** 2025-09-08

**Authors:** Katherine L. Ludorf, Renata H. Benjamin, Charles J. Shumate, Mark A. Canfield, Joanne Nguyen, A. J. Agopian

**Affiliations:** 1 Department of Epidemiology, University of Texas Health Science Center at Houston School of Public Health, Houston; 2 Birth Defects Epidemiology and Surveillance Branch, Texas Department of State Health Services, Austin; 3 Department of Epidemiology, University of Texas Health Science Center at Houston School of Public Health, Austin

## Abstract

**Importance:**

Trisomy 13 (T13) and trisomy 18 (T18) are chromosomal abnormalities with high mortality rates in the first year of life. Understanding differences in long-term survival between children with full vs mosaic or partial trisomy is crucial for prognosis and health care planning.

**Objective:**

To examine the differences in 10-year survival between children with full T13 and T18 vs those with mosaic or partial trisomy.

**Design, Setting, and Participants:**

This retrospective, population-based cohort study assessed liveborn infants with T13 and T18 in the Texas Birth Defects Registry (deliveries from January 1, 1999, to December 31, 2008). Follow-up was through December 31, 2018 (the last date available at the time of analyses) to allow for 10 years of follow-up for all infants. All analyses were conducted from January 1, 2022, to December 31, 2024.

**Exposures:**

Cytogenetic status (full trisomy vs mosaic or partial trisomy).

**Main Outcomes and Measures:**

The primary outcome was survival to 10 years of age, assessed using Kaplan-Meier survival estimates. The association between cytogenetic status and mortality by 10 years of age was assessed using Cox proportional hazards regression to generate hazard ratios (HRs). Population attributable fraction was calculated to determine the percentage of survival attributable to mosaic or partial trisomy status.

**Results:**

The study cohort included 798 infants (463 female infants [58.0%]; mean [SD] maternal age, 30.9 [8.0] years) with T13 (n = 295) or T18 (n = 503). Among all cases with T13, 25 infants (8.5%; 95% CI, 5.5%-12.3%) survived to 10 years of age. Similarly, among all infants with T18, 43 (8.6%; 95% CI, 6.3%-11.3%) survived to 10 years of age. Kaplan-Meier survival estimates to 10 years of age were statistically significantly higher among children with mosaic or partial trisomy (13 [25.0%] and 14 [43.8%], respectively) compared with full trisomy (12 [4.9%] and 29 [6.6%], respectively) (both *P* < .001). Infants with full trisomy had statistically significantly increased 10-year mortality hazards compared with those with mosaic or partial trisomy for both T13 (HR, 2.00; 95% CI, 1.42-2.82) and T18 (HR, 3.34; 95% CI, 2.08-5.38). The results of the calculated proportion of 10-year survival due to the presence of nonfull trisomy status (population attributable fraction) was 41.7% for children with T13 and 27.9% for children with T18.

**Conclusions and Relevance:**

The findings of this cohort study of infants with T13 and T18 support differences in long-term survival based on cytogenetic status and emphasize the need to potentially reassess the context of these conditions generally being considered incompatible with life, particularly for those with mosaic trisomies. These findings offer context surrounding treatment decisions, such as withholding interventions, for affected infants in the future.

## Key Points

**Question:**

What are the differences in survival to 10 years of age between children with full trisomy 13 or 18 vs those with mosaic or partial trisomy?

**Findings:**

In this cohort study of 798 infants with trisomy 13 and 18, survival to 10 years of age was statistically significantly higher among those with mosaic or partial trisomy compared with full trisomy.

**Meaning:**

These findings suggest that survival to 10 years of age may be relatively common among those with mosaicism or partial trisomy, and families of affected children may benefit from tailored prognostic counseling and care.

## Teaser

This cohort study of infants with trisomy 13 and 18 assesses survival differences in 10-year survival by full vs mosaic or partial trisomy status.

## Introduction

Trisomy 13 (T13; also known as Patau syndrome) and trisomy 18 (T18; also known as Edwards syndrome) are chromosomal abnormalities that occur in approximately 1 in 10 000 and 1 in 50 00 live births, respectively, positioning them as the most prevalent autosomal trisomies after trisomy 21.^1^ Affected individuals have multiple congenital anomalies (eg, congenital heart defects [CHDs]) and high mortality rates, with most dying within the first year.^1,2^ Infants with these conditions experience a broad array of anomalies that can affect nearly every organ system,^3,4^ and despite improvements in medical care over time that have positively affected survival (eg, surgical management for cardiac defects), the prognosis for children with T13 and T18 remains poor.^1,2,5^

Given recent changes in health care policies, a potential increase in the number of liveborn infants affected by conditions such as T13 and T18 is expected in many states in the US.^6^ Thus, because the medical community at large may encounter these conditions more frequently among liveborn infants in the future, enhancing our understanding of the factors affecting long-term survival is critical. Ethical dilemmas in decision-making frequently arise due to the severe and life-limiting nature of these conditions, particularly when determining the appropriate timing and extent of care for these infants.^7^ Controversy persists regarding when and how to intervene (eg, offering corrective cardiac surgical procedures) because prognoses are often shaped by expectations surrounding the severity of the condition.^8^

Insights into how cytogenetic trisomy status impacts health outcomes could inform the development of more targeted health care strategies. Most instances of T13 and T18 involve full trisomies,^9,10^ whereby essentially every cell in the body contains 3 entire copies of the chromosome. However, some cases exhibit mosaic trisomies, in which some cells (eg, in certain tissues or organ systems) contain 3 copies and others contain 2, or partial trisomies, whereby every cell contains 3 copies of only part of the chromosome but 2 copies of the rest of the chromosome. These trisomies can therefore be classified as full trisomies or nonfull (mosaic or partial) trisomies. Anecdotal evidence suggests those with full T13 or T18 may experience more severe phenotypes and worse outcomes, particularly short-term outcomes, such as survival to 1 year of age.^11^ Case reports and case series have reported on the association between cytogenetic trisomy status and long-term survival^9,10,11^; however, reports focusing on this subset of trisomy type at the population level is lacking. As many survival estimates are generated among the overall aggregate group of all cases rather than by cytogenetic status,^1,5,12,13^ counseling and decision-making for children with mosaic and partial trisomies may be made based on survival estimates not accurately reflective of long-term outcomes among those with nonfull trisomies. Heterogeneity in severity of how these conditions present may necessitate closer consideration of cytogenetic information on a larger scale. Thus, this study assessed differences in 10-year survival rates by full vs mosaic or partial trisomy status among children with T13 and T18 in a large, population-based sample.

## Methods

The protocol for this study was approved by the institutional review boards of University of Texas Health Science Center at Houston and the Texas Department of State Health Services. The staff of the Texas Birth Defects Registry (TBDR) has legislative authority to collect the TBDR data on all deliveries in Texas without individual consent.^14^ This study followed the Strengthening the Reporting of Observational Studies in Epidemiology (STROBE) reporting guideline for cohort studies.^15^

### Study Cases

Study cases were selected from the TBDR, a statewide program for monitoring birth defects. The TBDR is operated by the Birth Defects Epidemiology and Surveillance Branch of the Texas Department of State Health Services. Registry staff conduct active surveillance to identify individuals with birth defects by examining medical records at all health care facilities across the state where deliveries take place, such as hospitals and birthing centers. To be included in the registry, individuals must have had a diagnosis of a chromosomal abnormality or structural birth defect prenatally (with cytogenetic confirmation) or within the 12 months after delivery. A 6-digit British Pediatric Association (BPA) code modified by the Centers for Disease Control and Prevention is then assigned to each diagnosed defect for every eligible case.^16^ Records from the TBDR are routinely linked to state vital records data (birth, fetal death, and death certificates).

The study population included liveborn infants diagnosed with T13 (BPA codes 758.100-758.190) or T18 (BPA codes 758.200-758.290) delivered between January 1, 1999, and December 31, 2008, to allow for 10 years of follow-up for all infants, through December 31, 2018 (the last date available at the time of analyses). The included BPA codes were representative of the cytogenetic status of the trisomy present for an infant. For example, a BPA code of 758.240 indicated an infant was classified as having mosaic T18, whereas a BPA code of 758.200 indicated full or complete T18. Pregnancy terminations and spontaneous fetal deaths were considered ineligible. We also restricted cases to those determined by the TBDR to have a definite (as opposed to probable or possible) T13 or T18 diagnosis. For eligible cases, relevant BPA codes, case comments, and available cytogenetic information (eg, karyotype results) were examined to define the cytogenetic trisomy status for each infant as full trisomy or nonfull trisomy (ie, mosaic trisomy or partial trisomy). Available cytogenetic information was surveilled from medical records, which can include, for instance, results from a traditional cytogenetic karyotype, chromosomal microarray analysis, fluorescence in situ hybridization, or other diagnostic tools ordered by presiding clinicians. Partial trisomies included all translocation trisomies. Given relatively small numbers, mosaic and partial trisomies were combined into the same analytic group for analysis (nonfull trisomies). Infants determined to have co-occurring trisomy 21 or other syndromes were excluded (n < 5).

Data on infant and maternal characteristics (eg, demographic information) were obtained from medical records and/or vital records from the Center for Health Statistics at the Texas Department of State Health Services. Mortality status for eligible infants was determined through the use of Texas vital statistics data, which included the presence or absence of a linked death certificate, similar vital records from most other US states provided to the registry under state reciprocity agreements, and abstracted medical records. Survival time was calculated as the number of days alive (by subtracting the date of birth from the date of death). Deaths occurring outside Texas in states with which Texas had an interagency agreement were included in estimates (via the National Association for Public Health Statistics and Information Systems). Prior research in the TBDR has found that linking to additional resources, such as the National Death Index, to assess 5-year mortality, yielded few additional deaths beyond those already captured by the registry.^17^ All individuals lacking documented death in medical records or a connected death certificate were presumed to be living.^18^ Among those without death records, the date of birth was subtracted from the censoring date (ie, 10th birthday or December 31, 2018, the last available date for linked vital records during the analysis period).

### Statistical Analysis

All analyses were conducted separately for infants with T13 and T18. Numbers (percentages) for demographic characteristics were reported by T13 or T18 designation. Kaplan-Meier (KM) estimates were used to describe survival up to 10 years of age by cytogenetic status (full vs mosaic or partial trisomy) and used log-rank tests to compare survival distributions between the groups. To assess the association between the cytogenetic trisomy status (full vs nonfull) and mortality by 10 years of age, crude hazard ratios (HRs) were calculated with 95% CIs using Cox proportional hazards regression. To evaluate whether the proportional hazards assumption was met, we assessed time-dependent interaction terms.

These analyses were repeated after univariate adjustment for potential confounding variables: maternal age group (<20 years, 20-24 years, 25-29 years, 30-34 years, and ≥35 years) and maternal race and ethnicity group (Hispanic, non-Hispanic Black, non-Hispanic White, and other non-Hispanic, including American Indian or Alaska Native, Asian, Pacific Islander, and other specified groups). Maternal race and ethnicity group was ascertained from vital record data within the registry. To assess the effect of certain covariates on survival, we additionally performed Cox proportional hazards regression analyses post hoc and separately adjusted for birth year cohort, preterm birth, low birth weight, small for gestational age, presence of a CHD, and presence of a critical CHD. Critical CHDs are typically defined as heart defects requiring surgical intervention in the first year of life^19^; however, their specific impacts among infants with T13 and T18 remain not well understood. We classified CCHD status based on previously published criteria.^20^

Due to the relatively small number of deaths among infants with nonfull trisomy, there were insufficient numbers to conduct multivariable adjustments within this and other subgroups. Finally, we calculated the population attributable fraction (PAF) for survival percentage attributable specifically to nonfull trisomy status, using the observed risk ratios among all infants with T13 and T18.

In our secondary analyses, we found that none of our adjusted estimates from univariate assessment of the aforementioned 7 variables resulted in a meaningful change in effect estimate. Thus, we also conducted a secondary analysis to examine the strength of association an unmeasured confounder would need to have with the exposure and outcome to impact our results (ie, attenuate HR 95% CI estimates to a range that would include 1.0 or attenuate HR point estimates to 1.0). These estimates were generated using the E-value calculator^21^ by inputting the generated HRs and 95% CI estimates from the main analyses. All analyses were conducted using SAS software, version 9.4 (SAS Institute Inc) from January 1, 2022, to December 31, 2024.

## Results

The final sample of eligible liveborn infants from the TBDR between 1999 and 2008 included 798 infants (463 female infants [58.0%]; 403 [51.0%] Hispanic, 114 [14.3%] non-Hispanic Black, 245 [30.7%] non-Hispanic White, and 32 [4.1%] other non-Hispanic; mean [SD] maternal age, 30.9 [8.0] years), including 295 infants with T13 and 503 infants with T18 (Table 1). Overall, 52 infants with T13 (17.6%) and 32 infants with T18 (6.4%) received a nonfull trisomy designation (mosaic or partial trisomy). For both groups, 558 infants (69.9%) had cytogenetic information (eg, karyotype) available, with the remaining 240 (30.1%) receiving a diagnosis of T13 or T18 based on review of their medical records by registry staff. Demographic characteristics among infants with T13 and T18 were tabulated (Table 1). Of note, 80 infants (27.9%) with T13 and 214 (43.5%) of those with T18 were born to women 35 years of age or older. Additionally, 144 infants (48.8%) with T13 and 263 (52.3%) with T18 were born to Hispanic women.

**Table 1.** Characteristics of the Liveborn Infants With Trisomy 13 and 18^a^^,^^b^

Characteristic	No. (%) of infants	
	Trisomy 13 (n = 295)	Trisomy 18 (n = 503)
Infant sex		
Female	141 (48.0)	322 (64.0)
Male	153 (52.0)	181 (36.0)
Maternal race and ethnicity		
Hispanic	144 (48.8)	263 (52.3)
Non-Hispanic		
Black	47 (15.9)	67 (13.3)
White	94 (31.9)	151 (30.0)
Other^c^	10 (3.4)	22 (4.4)
Maternal educational level		
Less than high school	87 (31.5)	158 (33.2)
High school	79 (28.6)	121 (25.4)
Greater than high school	110 (39.9)	197 (41.4)
Maternal age, y		
<20	34 (11.9)	39 (7.9)
20-24	65 (22.7)	77 (15.7)
25-29	47 (16.4)	76 (15.5)
30-34	61 (21.3)	86 (17.5)
≥35	80 (27.9)	214 (43.5)
Tobacco cigarette use		
Smoker	18 (6.3)	20 (4.1)
Nonsmoker	269 (93.7)	470 (95.9)
Previous live births		
No	87 (31.6)	127 (26.5)
Yes	188 (68.4)	353 (73.5)
Birth cohort group		
1999-2004	178 (60.3)	256 (50.9)
2005-2008	117 (39.7)	247 (49.1)
Preterm birth status		
≥37-wk gestation	142 (48.1)	275 (54.7)
<37-wk gestation	153 (51.9)	228 (45.3)
Low birth weight		
≥2500 g	112 (38.1)	46 (9.2)
<2500 g	182 (61.9)	456 (90.8)
Small for gestational age		
No	150 (54.0)	55 (11.7)
Yes	128 (46.0)	415 (88.3)
Congenital heart defect		
Absent	123 (41.7)	165 (32.8)
Present	172 (58.3)	338 (67.2)
Critical congenital heart defect		
Absent	237 (80.3)	405 (80.5)
Present	58 (19.7)	98 (19.5)

^a^ Data are from the Texas Birth Defects Registry, 1999 to 2008.

^b^ Numbers may not total due to missing data.

^c^ Other includes American Indian or Alaska Native, Asian, Pacific Islander, and other specified groups.

Among all cases with T13, 25 infants (8.5%; 95% CI, 5.5%-12.3%) survived to 10 years of age. Similarly, among all infants with T18, 43 (8.6%; 95% CI, 6.3%-11.3%) survived to 10 years of age. However, a much higher proportion of those with nonfull trisomy survived to 10 years of age compared with the full group (13 [25.0%] for T13 and 14 [43.8%] for T18) (Table 2). In KM survival curves, for T13, most deaths occurred within 2 months of birth, and this rate declined rapidly before reaching a steadier plateau (Figure, A), although, for T18, most deaths were spread over the first year overall, reaching a plateau more slowly (Figure, B). KM survival estimates to 10 years of age were statistically significantly higher among children with mosaic or partial trisomy (13 [25.0%] and 14 [43.8%], respectively) compared with full trisomy (12 [4.9%] and 29 [6.6%], respectively) (both *P* < .001) (Figure, A-B).

**Table 2.** PAFs for the Association Between Mosaic or Partial vs Full Trisomy Status and Survival to 10 Years of Age Among Liveborn Infants With Trisomy 13 and 18^a^

Trisomy status	PAF, %^c^	Survival to 10 years of age, No. (%)^b^	
		No	Yes
Trisomy 13			
Nonfull (n = 52)^d^	41.7	39 (75.0)	13 (25.0)
Full (n = 243)	Reference	231 (95.1)	12 (4.9)
Trisomy 18			
Nonfull (n = 32)^d^	27.9	18 (56.3)	14 (43.8)
Full (n = 468)	Reference	439 (93.4)	29 (6.6)

Abbreviation: PAF, population attributable fraction.

^a^ Data are from the Texas Birth Defects Registry, 1999 to 2008.

^b^ Percentages are calculated by the row.

^c^ PAF = [Prevalence of the Exposure (Relative Risk – 1)]/[Prevalence of the Exposure (Relative Risk – 1) + 1].

^d^ Included mosaic and partial trisomies.

**Figure.**
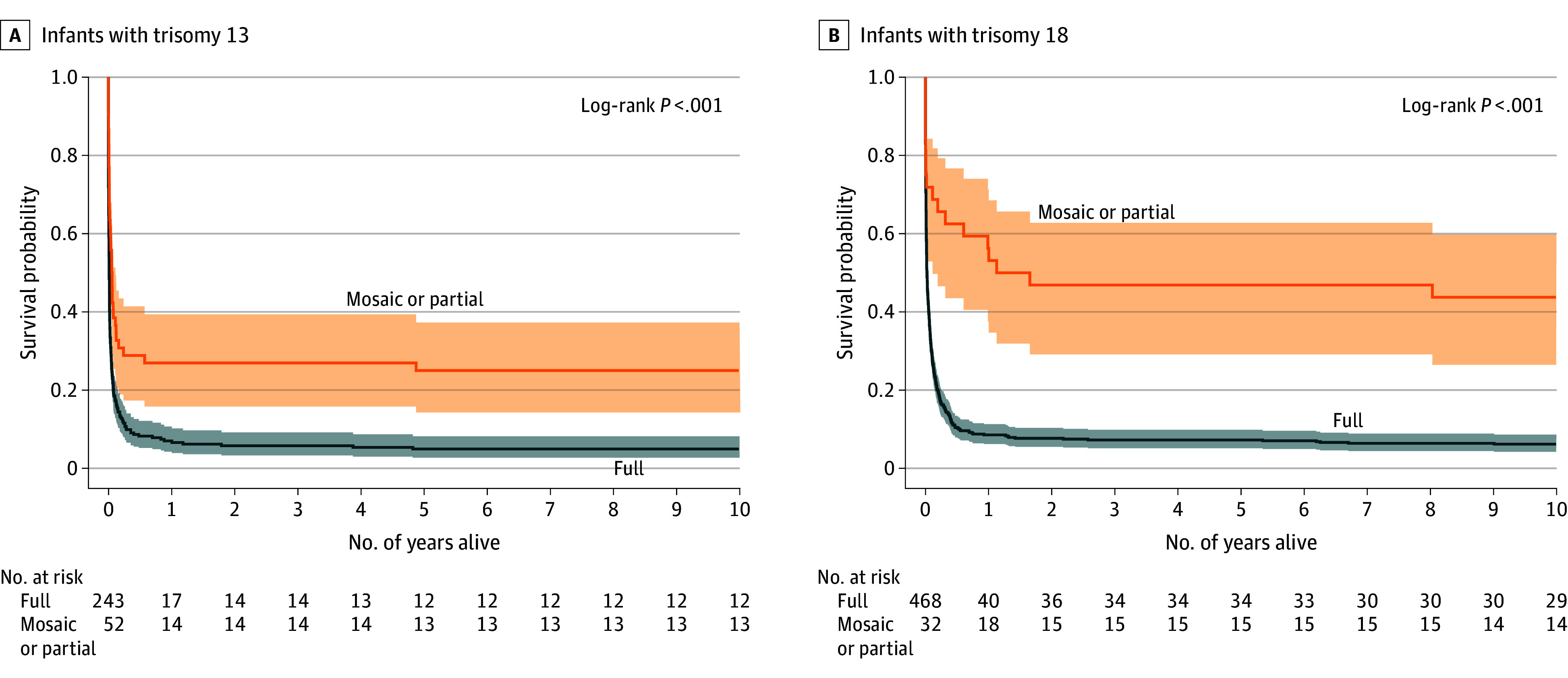
Ten-Year Survival Estimates by Mosaic or Partial Trisomy Status Among Liveborn Infants With Trisomy 13 and 18, Texas Birth Defects Registry, 1999 to 2008 Shaded areas indicate 95% CIs.

In the main results, infants with full trisomy had statistically significantly increased 10-year mortality hazards compared with those with mosaic or partial trisomy for both T13 (HR, 2.00; 95% CI, 1.42-2.82) and T18 (HR, 3.34; 95% CI, 2.08-5.38) (Table 3). Individually adjusting for maternal age group, maternal race and ethnicity group, birth year cohort, preterm birth, low birth weight, small for gestational age, presence of a CHD, and presence of a critical CHD in univariate analyses yielded largely similar results to the main analyses across both children with T13 and T18 (Table 3). The results of the calculated proportion of 10-year survival due to the presence of nonfull trisomy status (PAF) was 41.7% for children with T13 and 27.9% for children with T18.

**Table 3.** Unadjusted and Univariate Adjusted Survival Estimates for the Association Between Mosaic or Partial Trisomy Status and Survival to 10 Years of Age Among Children With Trisomy 13 or Trisomy 18^a^

Adjustment	HR (95% CI)	
	Trisomy 13	Trisomy 18
Unadjusted	2.00 (1.42-2.82)	3.34 (2.08-5.38)
Adjusted^b^		
Maternal age	2.07 (1.46-2.94)	3.63 (2.24-5.87)
Maternal race and ethnicity	2.04 (1.44-2.87)	3.43 (2.13-5.52)
Birth year group	1.96 (1.39-2.77)	3.38 (2.10-5.45)
Preterm birth	1.98 (1.41-2.79)	3.34 (2.08-5.38)
Low birth weight	1.95 (1.38-2.75)	2.82 (1.71-4.64)
Small for gestational age	2.00 (1.40-2.86)	3.58 (2.13-6.01)
Congenital heart defect	2.02 (1.43-2.84)	4.01 (2.48-6.49)
Critical congenital heart defect	1.53 (1.02-2.30)	2.84 (1.51-5.34)

Abbreviation: HR, hazard ratio.

^a^ Data are from the Texas Birth Defects Registry, 1999 to 2008.

^b^ Adjusted for each single covariate separately due to insufficient numbers to adjust for all covariates simultaneously.

Sensitivity analyses yielded E-values of 2.61 for infants with T13 and 3.98 for infants with T18, corresponding to the magnitudes of association an unmeasured confounder would need to have with the exposure (cytogenetic trisomy status) and outcome (mortality) to result in attenuation of the observed HRs to 1.0. The E-values corresponding to magnitudes of association an unmeasured confounder would need to reduce the lower limit of the 95% CI to 1.0 (ie, a nonsignificant HR) were calculated to be 1.87 and 2.70 for T13 and T18, respectively.

## Discussion

Our findings suggest that children with nonfull T13 and T18 have an approximately 2- and 3-fold higher chance of surviving to 10 years of age, respectively, compared with those with full trisomy. For conditions often deemed to be incompatible with life, there were relatively high proportions of infants with nonfull T13 (13 [25.0%]) and T18 (14 [43.8%]) surviving to at least 10 years of age. Observed results may prompt reconsideration of interventions previously viewed as prolonging life artificially or potentially diminishing quality of life among patients with otherwise fatal defects.^22^ Recent guidelines from the American Association for Thoracic Surgery have suggested that surgical intervention for liveborn infants with T13 and T18 should be evaluated at the individual level rather than the diagnostic level because these cases are often complex and multifactorial.^8^ Additionally, quality-of-life metrics for surviving individuals have been reported as positive by their families,^23^ highlighting the possibility that providing life-extending care may be prioritized differently within specific family dynamics. Thus, our results may support the framework for reevaluating the ethical considerations over implementing vs withholding medical interventions for these individuals and may serve to inform decision-making in the management of affected pregnancies, especially considering the reported survival trajectories among those with mosaic or partial trisomies. For instance, many families and physicians might consider the expected greater than 40% likelihood of survival to 10 years of age among children with mosaic or partial T13 to be compelling for certain medical interventions.

Given that, to our knowledge, no prior studies have reported on long-term (eg, to 10 years of age) survival for children with T13 and T18 by cytogenetic trisomy status, direct comparison of our association results with prior epidemiologic literature cannot be made. However, our results seem to generally align with a smaller association study among individuals who underwent surgery (eg, 17 patients with nonfull T13) that reported that infants who survived to older than 1 year were statistically significantly more likely to have nonfull T13 or T18 compared with those who died by 1 year of age (26.5% survival to ≥1 year of age).^24^ Our 10-year survival proportions among all infants with (full and mosaic or partial) T13 (25 [8.5%]) and T18 (43 [8.6%]) also seem fairly consistent with those from 2 population-based studies^5,24^ that reported survival estimates to 10 years of age for all infants (10.8%-12.9% for T13 and 8.0%-9.8% for T18) (eTable in Supplement 1), but we are unaware of similar estimates for those with nonfull T13 or T18.

Examination of long-term survival trends among neonates, infants, and children with mosaicism at the population level remain less well described. Although full trisomies generally manifest more severe clinical phenotypes than nonfull trisomies, population-based comparisons are crucial to confirm and characterize these differences at scale, especially given the rarity of nonfull T13 and T18. It is also unclear how nonfull T13 and T18 specifically impact developmental mechanisms, resulting in variable phenotypic severity and complexity among individual patients.^25^ These complexities underscore the need for more comprehensive research to better understand the impact of mosaicism or partial trisomy on disease progression and management strategies.

Currently, the mechanisms underlying mosaicism are theorized to occur for several reasons. One is through incomplete trisomic rescue, where a trisomic cell loses a chromosome during division, creating a diploid cell line. Given that the frequency of trisomic rescue in cells is tissue type dependent,^26^ certain organs and body systems might be more susceptible to mosaicism than others, which might translate to a variable range of incremental to substantial reduction in phenotypic severity and complexity, depending on the organ or body system. Somatic mosaicism can also arise from errors in later-stage chromosomal segregation, resulting in differing proportions of affected cells across tissues.^27,28,29^ The clinical presentation in mosaic trisomy, therefore, can vary considerably among individuals, potentially due to the presence of cell lines with a normal chromosome count providing some protection at the organ level against adverse effects of the additional chromosome in other cell lines.

Diverse clinical presentation means prenatal diagnosis may offer an opportunity for early intervention among those with mosaicism. Early detection of trisomy type (full vs nonfull trisomy) may aid in decisions regarding the medical necessity and timing of interventions, planning for potential comorbidities (eg, heart defects), and providing additional prognostic information to parents. Intervention at an earlier stage may impact long-term health and survival outcomes for individuals with mosaicism or partial trisomy.

### Strengths and Limitations

Strengths of our analyses include access to nearly 2 decades of statewide data from a large population-based registry through the TBDR, which represents one of the largest sources of population-based birth defect registry data in the US and allowed for evaluation of long-term survival. These data allowed for differentiation between those with full vs nonfull trisomies as well as exclusion of potential infants with additional co-occurring chromosomal anomalies (eg, trisomy 21).

Our study also had limitations. The primary limiting factor was that, despite our large population and long study period, the rarity of T13 and T18 and inherent heterogeneity of the conditions meant evaluations of comparisons were limited; this rarity also precluded the use of multivariable modeling in our main analysis. Additionally, we chose to combine all partial trisomies because our sample did not include enough eligible cases to further parse out cytogenetic differences.

Incomplete ascertainment of death was also a potential limitation of our analyses; a small number of deaths may have been missed due to inaccurate or missing data, creating difficulties in linking registry cases and death certificates. However, the impact of any missing deaths was likely minimal because previous work in the TBDR has demonstrated that enhancing data with national registry information provided a relatively small number of supplemental cases.

We were additionally limited by lack of availability of data on health care utilization, medical and surgical management, or clinical trajectories of included infants. Relevant health data such as these offer a more comprehensive understanding of the outcomes and factors influencing the health of these children and should be considered in conjunction with genetic factors in any future work. Consistent with this, our PAF calculation demonstrated more than 50% of deaths in both groups could not be attributed to cytogenetic status alone, highlighting the role of other factors in infant outcomes. However, our univariate adjustment for maternal age, preterm birth, small for gestational age, low birth weight, and presence of a heart defect largely did not meaningfully impact the HR for T13 or T18 estimates, and our sensitivity analyses suggested that any unmeasured confounders would need to have very large magnitudes of effect with the exposure and outcome (eg, >2.97-3.95) to strongly impact our overall interpretation; therefore, it is unlikely that unmeasured confounding could completely explain away the observed associations. Larger pooled samples such as those that could be amassed from the National Birth Defects Prevention Network or the International Clearinghouse for Birth Defects Research and Prevention may help to facilitate these types of additional analyses, although differences across surveillance systems in documentation and coding of cytogenetic details may pose additional challenges in pooling data.

## Conclusions

We found that individuals with mosaic or partial T13 or T18 compared with those with full trisomy had a greater likelihood of survival to 10 years of age. However, the results also highlight the need to identify additional factors associated with long-term survival among infants with T13 and T18 because less than 50% of survival was attributable to nonfull trisomy status. Additional studies examining the impact of early interventions, such as palliative care or surgical procedures, and clinically relevant factors, such as small-for-gestational-age status, could offer valuable insights into understanding and ultimately improving survival outcomes for affected children. Type-dependent survival demonstrated in our results also emphasizes the need to prioritize cytogenetic classification for development of more tailored care among livebirth infants affected with these anomalies in the US moving forward.

## Notes

Supplement 1. **eTable.** Select Publications Examining Long-Term Survival Among Infants With Trisomy 13 and Trisomy 18
eReferences

Supplement 2.
Data Sharing Statement

## References

[1] Goel N, Morris JK, Tucker D, . Trisomy 13 and 18-prevalence and mortality: a multi-registry population based analysis. Am J Med Genet A. 2019;179(12):2382-2392. doi:10.1002/ajmg.a.61365 31566869 PMC6848757

[2] Springett A, Wellesley D, Greenlees R, . Congenital anomalies associated with trisomy 18 or trisomy 13: a registry-based study in 16 European countries, 2000-2011. Am J Med Genet A. 2015;167A(12):3062-3069. doi:10.1002/ajmg.a.37355 26347425

[3] Rosa RF, Rosa RC, Zen PR, Graziadio C, Paskulin GA. Trisomy 18: review of the clinical, etiologic, prognostic, and ethical aspects. Rev Paul Pediatr. 2013;31(1):111-120. doi:10.1590/S0103-05822013000100018 23703053

[4] Diaz D, Benjamin RH, Navarro Sanchez ML, . Patterns of congenital anomalies among individuals with trisomy 13 in Texas. Am J Med Genet A. 2021;185(6):1787-1793. doi:10.1002/ajmg.a.62175 33749998 PMC8193718

[5] Glinianaia SV, Rankin J, Tan J, . Ten-year survival of children with trisomy 13 or trisomy 18: a multi-registry European cohort study. Arch Dis Child. 2023;108(6):461-467. doi:10.1136/archdischild-2022-325068 36882305

[6] Gemmill A, Margerison CE, Stuart EA, Bell SO. Infant deaths after Texas’ 2021 ban on abortion in early pregnancy. JAMA Pediatr. 2024;178(8):784-791. doi:10.1001/jamapediatrics.2024.0885 38913344 PMC11197445

[7] Kosiv KA, Mercurio MR, Carey JC. The common trisomy syndromes, their cardiac implications, and ethical considerations in care. Curr Opin Pediatr. 2023;35(5):531-537. doi:10.1097/MOP.0000000000001278 37551160

[8] St Louis JD, Bhat A, Carey JC, . The American Association for Thoracic Surgery (AATS) 2023 Expert Consensus Document: recommendation for the care of children with trisomy 13 or trisomy 18 and a congenital heart defect. J Thorac Cardiovasc Surg. 2024;167(5):1519-1532. doi:10.1016/j.jtcvs.2023.11.054 38284966

[9] Bugge M, Collins A, Petersen MB, . Non-disjunction of chromosome 18. Hum Mol Genet. 1998;7(4):661-669. doi:10.1093/hmg/7.4.661 9499419

[10] Cammarata-Scalisi F, Araque D, Ramírez R, Guaran L, Silva GD. Trisomy 13 mosaicism. Boletin Med Hosp Infant Mexico. 2019;76(5):246-250. doi:10.24875/BMHIM.19000003 31536039

[11] Wu J, Springett A, Morris JK. Survival of trisomy 18 (Edwards syndrome) and trisomy 13 (Patau syndrome) in England and Wales: 2004-2011. Am J Med Genet A. 2013;161A(10):2512-2518. doi:10.1002/ajmg.a.36127 23949924

[12] Meyer RE, Liu G, Gilboa SM, ; National Birth Defects Prevention Network. Survival of children with trisomy 13 and trisomy 18: a multi-state population-based study. Am J Med Genet A. 2016;170A(4):825-837. doi:10.1002/ajmg.a.37495 26663415 PMC4898882

[13] Wang Y, Hu J, Druschel CM, Kirby RS. Twenty-five-year survival of children with birth defects in New York State: a population-based study. Birth Defects Res A Clin Mol Teratol. 2011;91(12):995-1003. doi:10.1002/bdra.22858 21960515

[14] Texas Health and Human Services. Texas Administrative Code. Texas Health and Human Safety Code. Title 25, Part 1, Chapter 37, Subchapter P, Rules 37.301-37.306. Accessed July 30, 2025. https://texas-sos.appianportalsgov.com/rules-and-meetings?chapter=37&interface=VIEW_TAC&part=1&subchapter=P&title=25

[15] von Elm E, Altman DG, Egger M, Pocock SJ, Gøtzsche PC, Vandenbroucke JP; STROBE Initiative. The Strengthening the Reporting of Observational Studies in Epidemiology (STROBE) statement: guidelines for reporting observational studies. Ann Intern Med. 2007;147(8):573-577. doi:10.7326/0003-4819-147-8-200710160-00010 17938396

[16] Rasmussen SA, Olney RS, Holmes LB, Lin AE, Keppler-Noreuil KM, Moore CA; National Birth Defects Prevention Study. Guidelines for case classification for the National Birth Defects Prevention Study. Birth Defects Res A Clin Mol Teratol. 2003;67(3):193-201. doi:10.1002/bdra.10012 12797461

[17] Marengo LK, Hoyt AT, Canfield MA. The utility of the National Death Index as a supplemental data source in ascertaining 5-year mortality among Texas heterotaxy cases. J Registry Manag. 2014;41(1):4-6. 24893181

[18] Benjamin RH, Lopez A, Mitchell LE, . Mortality by mode of delivery among infants with spina bifida in Texas. Birth Defects Res. 2019;111(19):1543-1550. doi:10.1002/bdr2.1608 31642615 PMC7741424

[19] Mahle WT, Newburger JW, Matherne GP, ; American Heart Association Congenital Heart Defects Committee of the Council on Cardiovascular Disease in the Young, Council on Cardiovascular Nursing, and Interdisciplinary Council on Quality of Care and Outcomes Research; American Academy of Pediatrics Section on Cardiology And Cardiac Surgery; Committee On Fetus And Newborn. Role of pulse oximetry in examining newborns for congenital heart disease: a scientific statement from the AHA and AAP. Pediatrics. 2009;124(2):823-836. doi:10.1542/peds.2009-1397 19581259

[20] Pace ND, Oster ME, Forestieri NE, Enright D, Knight J, Meyer RE. Sociodemographic factors and survival of infants with congenital heart defects. Pediatrics. 2018;142(3):e20180302. doi:10.1542/peds.2018-0302 30111552

[21] Mathur MB, Ding P, Riddell CA, VanderWeele TJ. Website and R package for computing E-values. Epidemiology. 2018;29(5):e45-e47 29912013 10.1097/EDE.0000000000000864 PMC6066405

[22] Pallotto I, Lantos JD. Treatment decisions for babies with trisomy 13 and 18. HEC Forum. 2017;29(3):213-222. doi:10.1007/s10730-017-9319-2 28365826

[23] Janvier A, Farlow B, Wilfond BS. The experience of families with children with trisomy 13 and 18 in social networks. Pediatrics. 2012;130(2):293-298. doi:10.1542/peds.2012-0151 22826570

[24] Nelson KE, Rosella LC, Mahant S, Guttmann A. Survival and surgical interventions for children with trisomy 13 and 18. JAMA. 2016;316(4):420-428. doi:10.1001/jama.2016.9819 27458947

[25] Griffith CB, Vance GH, Weaver DD. Phenotypic variability in trisomy 13 mosaicism: two new patients and literature review. Am J Med Genet A. 2009;149A(6):1346-1358. doi:10.1002/ajmg.a.32883 19449431

[26] Inoue M, Kajiwara K, Yamaguchi A, . Autonomous trisomic rescue of Down syndrome cells. Lab Invest. 2019;99(6):885-897. doi:10.1038/s41374-019-0230-0 30760866 PMC6760570

[27] Papavassiliou P, Charalsawadi C, Rafferty K, Jackson-Cook C. Mosaicism for trisomy 21: a review. Am J Med Genet A. 2015;167A(1):26-39. doi:10.1002/ajmg.a.36861 25412855

[28] Papavassiliou P, York TP, Gursoy N, . The phenotype of persons having mosaicism for trisomy 21/Down syndrome reflects the percentage of trisomic cells present in different tissues. Am J Med Genet A. 2009;149A(4):573-583. doi:10.1002/ajmg.a.32729 19291777 PMC3707311

[29] Yokoyama Y, Narahara K, Kamada M, Tsuji K, Seino Y. Tissue-specific mosaicism for trisomy 21 and congenital heart disease. J Pediatr. 1992;121(1):80-82. doi:10.1016/S0022-3476(05)82547-8 1385628

