# Supplementary material for: Long-Term Survival Among Children With Trisomy 13 and Trisomy 18 by Cytogenetic Status
Source: JAMA Netw Open. 2025 Sep 8;8(9):e2529885. doi: 10.1001/jamanetworkopen.2025.29885 (PMC12418129; doi:10.1001/jamanetworkopen.2025.29885)
Supplement: Supplement 1. — eTable. Select Publications Examining Long-Term Survival Among Infants With Trisomy 13 and Trisomy 18 eReferences [file jamanetwopen-e2529885-s001.pdf]

Supplemental Online Content

Ludorf KL, Benjamin RH, Shumate CJ, Canfield MA, Nguyen J, Agopian AJ. Long-term survival among children with trisomy 13 and trisomy 18 by cytogenetic status. *JAMA Netw Open*. 2025;8(9):e2529885. doi:10.1001/jamanetworkopen.2025.29885

eTable. Select Publications Examining Long-Term Survival Among Infants With Trisomy 13 and Trisomy 18

eReferences

This supplemental material has been provided by the authors to give readers additional information about their work.

© 2025 Ludorf KL et al. *JAMA Network Open.*

eTable. Select Publications Examining Long-Term Survival Among Infants With Trisomy 13 and Trisomy 18

	**Trisomy 13**				**Trisomy 18**				
	**Mosaic or partial trisomy (n)**	**Total (n)**	**5-year survival (%)**	**10-year survival (%)**	**Mosaic or partial trisomy (n)**	**Total (n)**	**5-year survival (%)**	**10-year survival (%)**	**Conducted population-based survival analysis by cytogenetic status for T13 and T18**
Case series/report[a]									
Ribeiro, R. R., et al.	-	-	-	-	-	1	-	-	No
Morán-Barroso, V. F., et al.	1	1	-	-	-	-	-	-	No
Imataka, G., et al.	-	1	-	-	-	-	-	-	No
González-del Angel, A., et al.	1	1	-	-	-	-	-	-	No
Ferreira de Souza, L.M., et al.	-	-	-	-	-	1	-	-	No
Hsu, H.-F. and Hou, J.-W.	2	13	-	-	-	-	-	-	No
Clinic-based study									
Peterson, J.K., et al.[b,c]	20	50	91.7[d]	91.7[d]	16	121	91.7[d]	91.7[d]	No
Population-based studies[e]				-					
Kato, N. et al.	-	1,164	-	-	-	-	-	-	No
Goel, N., et al.	-	2,537	7.0	-	-	6,122	7.7	-	No
Glinianaia, S.V.,et al	-	252	16.1	10.8	-	602	10.0	8.0	No
Wang, Y., et al.	-	525	18.4	-	-	773	15.2	-	No
Schneuer, F.J., et al.	-	25	-	-	-	34	17.6	-	No
Meyer, R.E., et al.	-	693	9.7	-	-	1,113	12.3	-	No
Nelson, K.E., et al.[f]	17	174	15.0	12.9	18	254	11.0	9.8	No
Current Study	52	295	-	8.5	32	503	-	8.6	Yes

[a]≤15 subjects

[b]Evaluated infants from a clinic-based population who survived to 1 year of age and had undergone surgical intervention for a co-occurring CHD

[c]5-year or 10-year survival percentages presented by cytogenetic status

© 2025 Ludorf KL et al. *JAMA Network Open.*

[d]Combined T13 and T18 infant groups to calculate survival due to small cell counts (mosaic vs. full survival analysis)

[e]>15 subjects with observational research study design

[f]Evaluated full versus non-full trisomy with respect to survival to age one

© 2025 Ludorf KL et al. *JAMA Network Open.*

eReferences

Ferreira de Souza LM, Galvão EBMA, Júnior JPR, de Melo AN, Dias S. Long Survival of a Patient with Trisomy 18 and Dandy-Walker Syndrome. *Medicina (Kaunas, Lithuania)*. Jul 8 2019;55(7)doi:10.3390/medicina55070352

Glinianaia SV, Rankin J, Tan J, et al. Ten-year survival of children with trisomy 13 or trisomy 18: a multi-registry European cohort study. *Archives of disease in childhood*. Jun 2023;108(6):461-467. doi:10.1136/archdischild-2022-325068

Goel N, Morris JK, Tucker D, et al. Trisomy 13 and 18-Prevalence and mortality-A multi-registry population based analysis. *American journal of medical genetics Part A*. Dec 2019;179(12):2382-2392. doi:10.1002/ajmg.a.61365

González-del Angel A, Estandia-Ortega B, Gaviño-Vergara A, Sáez-de-Ocariz M, Velasco-Hernández Mde L, Salas-Labadía C. A patient with trisomy 13 mosaicism with an unusual skin pigmentary pattern and prolonged survival. *Pediatric dermatology*. Sep-Oct 2014;31(5):580-3. doi:10.1111/pde.12339

Hsu HF, Hou JW. Variable expressivity in Patau syndrome is not all related to trisomy 13 mosaicism. *American journal of medical genetics Part A*. Aug 1 2007;143a(15):1739-48. doi:10.1002/ajmg.a.31835

Imataka G, Hagisawa S, Nitta A, Hirabayashi H, Suzumura H, Arisaka O. Long-term survival of full trisomy 13 in a 14 year old male: a case report. *European review for medical and pharmacological sciences*. Mar 2016;20(5):919-22.

Kato N, Morisaki N, Moriichi A. Trends in the survival of patients with trisomy 13 from 1995 to 2021: A population study in Japan. *American journal of medical genetics Part A*. Sep 2024;194(9):e63710. doi:10.1002/ajmg.a.63710

Meyer RE, Liu G, Gilboa SM, et al. Survival of children with trisomy 13 and trisomy 18: A multi-state population-based study. *American journal of medical genetics Part A*. Apr 2016;170a(4):825-37. doi:10.1002/ajmg.a.37495

Morán-Barroso VF, Cervantes A, Rivera-Vega MDR, et al. Mosaic proximal trisomy 13q and regular trisomy 13 in a female patient with long survival: Involvement of an incomplete trisomic rescue and a chromothripsis event. *Molecular genetics & genomic medicine*. Sep 2021;9(9):e1762. doi:10.1002/mgg3.1762

Nelson KE, Rosella LC, Mahant S, Guttmann A. Survival and Surgical Interventions for Children With Trisomy 13 and 18. *JAMA*. 2016;316(4):420-428. doi:10.1001/jama.2016.9819

Peterson JK, Kochilas LK, Catton KG, Moller JH, Setty SP. Long-Term Outcomes of Children With Trisomy 13 and 18 After Congenital Heart Disease Interventions. *The Annals of thoracic surgery*. Jun 2017;103(6):1941-1949. doi:10.1016/j.athoracsur.2017.02.068

Ribeiro RR, dos Santos BM, Stuani AS, de Freitas AC, de Queiroz AM. Dental findings and dental care management in trisomy 18: case report of a 13-year-old "long-term survivor". *Special care in dentistry : official publication of the American Association of Hospital Dentists, the Academy of Dentistry for the Handicapped, and the American Society for Geriatric Dentistry*. Nov-Dec 2006;26(6):247-51. doi:10.1111/j.1754-4505.2006.tb01662.x

Schneuer FJ, Bell JC, Shand AW, Walker K, Badawi N, Nassar N. Five-year survival of infants with major congenital anomalies: a registry based study. *Acta Paediatrica*. 2019;108(11):2008-2018. doi:https://doi.org/10.1111/apa.14833

© 2025 Ludorf KL et al. *JAMA Network Open.*

Wang Y, Hu J, Druschel CM, Kirby RS. Twenty-five-year survival of children with birth defects in New York State: a population-based study. *Birth defects research Part A, Clinical and molecular teratology*. Dec 2011;91(12):995-1003. doi:10.1002/bdra.22858

© 2025 Ludorf KL et al. *JAMA Network Open.*
