# Supplementary material for: Long-Term Survival Among Children With Trisomy 13 and Trisomy 18 by Cytogenetic Status
Source: JAMA Netw Open. 2025 Sep 8;8(9):e2529885. doi: 10.1001/jamanetworkopen.2025.29885 (PMC12418129; doi:10.1001/jamanetworkopen.2025.29885)
Supplement: Supplement 2. — Data Sharing Statement [file jamanetwopen-e2529885-s002.pdf]

Data Sharing Statement

Ludorf. Long-Term Survival Among Children With Trisomy 13 and Trisomy 18 by Cytogenetic Status. *JAMA Netw Open*. Published September 04, 2025.
doi:10.1001/jamanetworkopen.2025.29885

Data
Data available: No

Additional Information
Explanation for why data not available: The data analyzed are not publicly available due to confidentiality of vital records.
